# Exploring subgroups of acceptance prediction for e-mental health among psychotherapists-in-training: a latent class analysis

**DOI:** 10.3389/fpsyt.2024.1296449

**Published:** 2024-03-14

**Authors:** Robert Staeck, Miriam Stüble, Marie Drüge

**Affiliations:** ^1^ University of Bern, Faculty of Medicine, Institute of Social and Preventive Medicine, Bern, Switzerland; ^2^ Graduate School for Health Sciences, University of Bern, Bern, Switzerland; ^3^ University Hospital of Child and Adolescent Psychiatry and Psychotherapy, University of Bern, Bern, Switzerland; ^4^ University of Zurich, Department of Psychology, Clinical Psychology with Focus on Psychotherapy Research, Zurich, Switzerland

**Keywords:** e-mental health, UTAUT, psychotherapists-in-training, acceptance, latent class analysis

## Abstract

**Theoretical background:**

Research of E-Mental Health (EMH) interventions remains a much-studied topic, as does its acceptance in different professional groups as psychotherapists-in-training (PiT). Acceptance among clinicians may vary and depend on several factors, including the characteristics of different EMH services and applications. Therefore, the aims of this study were to investigate the factors that predict acceptance of EMH among a sample of PiT using a latent class analysis. The study will 1) determine how many acceptance prediction classes can be distinguished and 2) describe classes and differences between classes based on their characteristics.

**Methods:**

A secondary analysis of a cross-sectional online survey was conducted. N = 216 PiT (88.4% female) participated. In the study, participants were asked to rate their acceptance of EMH, as operationalized by the Unified Theory of Acceptance and Use of Technology (UTAUT) model, along with its predictors, perceived barriers, perceived advantages and additional facilitators. Indicator variables for the LCA were eight items measuring the UTAUT-predictors.

**Results:**

Best model fit emerged for a two-class solution; the first class showed high levels on all UTAUT-predictors, the second class revealed moderate levels on the UTAUT-predictors.

**Conclusion:**

This study was able to show that two classes of individuals can be identified based on the UTAUT-predictors. Differences between the classes regarding Performance Expectancy and Effort Expectancy were found. Interestingly, the two classes differed in theoretical orientation but not in age or gender. Latent class analysis could help to identify subgroups and possible starting points to foster acceptance of EMH.

## 1 Introduction

The need for reliable and effective interventions to support mental health has grown rapidly, pushing the health system to its limits in many countries. One way to address this need is through E-Mental Health (EMH) interventions. EMH may be especially suited to address treatment barriers of underserved populations (e.g., rural areas, avoidance behavior due to shame/stigma) or waiting times. In recent years EMH has evolved and shown promising results in many studies in decreasing symptomatology (1), also in low- or middle-income countries (2). While evidence supports the effectiveness of EMH for mild to moderate mental health issues, caution is needed when considering its generalizability (e.g., selection bias, drop-out rates). Nevertheless, it remains of interest why EMH is rarely used in many countries (3). Therefore, barriers and facilitators to the integration of EMH in routine care have been discussed (4). One of the most frequent determinants of providing and receiving EMH in routine care is the acceptance of mental health care providers and patients (5). Interestingly, there is a systematic review suggesting that people with mental disorders and general practitioners have a more favorable view of EMH than psychotherapists, which poses a barrier to its implementation (6). This result was also found in more recent studies (7), however COVID-19 has accelerated the use of EMH (8, 9) and thus more positive attitudes towards online therapy were found (10). There are different theoretical models (e.g., Technology Acceptance Model, Unified Theory of Acceptance and Use of Technology - UTAUT) to operationalize acceptance of EMH f.e. as the intention to use technology such as EMH in general or a specific EMH application.

The UTAUT (11) contains four key constructs, namely Performance Expectancy (belief that using the system will enhance job performance), Effort Expectancy (expected ease of use), Social Influence (extent to which one believes significant others endorse using the new system), and Facilitating Conditions (organizational or technical resources exist for technology use) as predictors. This theory has been expanded in a variety of other studies using additional determinants (“knowledge of eHealth Interventions” cf. 12). Acceptance varied significantly between modalities (e.g., videoconferencing vs. unguided programs; cf. 13, 14). Also, UTAUT has been used in many studies which consider the perspective of the client (15, 16) as well as the perspective of the medical/psychological staff (12, 14, 17). Therefore, the UTAUT holds particular relevance regarding the acceptance of EMH in a medical context (12) in different target groups. Psychotherapists-in-Training (PiT) are an understudied and undervalued population which provide insight into the psychotherapy training. This bears relevance as the acceptance of EMH among PiT could influence the future of healthcare systems. However, to date, research on this specific group has been scarce, with only two studies utilizing the same dataset as the present study being published (14, 17): The overall acceptance of EMH among PiT, which was assessed on a 1-5 Likert scale and then categorized into the categories low (1–2.34), moderate (2.35–3.67), or high (3.68–5), can be described as moderate in N = 216 German-speaking PiT (14). This research also highlights the fact that Performance Expectancy, Social Influence and concerns about the therapeutic alliance determine EMH acceptance. Moreover, acceptance of psychotherapy via videoconference was rated the highest (M = 3.7, SD = 1.15) and acceptance of unguided programs was rated the lowest. In a secondary analysis interaction between the different application purposes (e.g. prevention, treatment addition, treatment substitute and aftercare) and different EMH modalities (e.g. telephone, videoconference, VR, unguided programs, guided programs) were analyzed (17). Although research has explored the general acceptance of EMH among PiT and other determinants of EMH (e.g., barriers, advantages) it remains unclear whether there are subgroups in this population and, if so, what characterizes those subgroups. So far, subgroup analysis using the UTAUT in a medical context has been conducted with dichotomized variables, employing a median-split (18) or using pre-existing categories (e.g., gender, no prior experience) (19, 20). Latent class analysis aims to achieve homogeneity within clusters while fostering heterogeneity between clusters (21). The number of distinct classes is not defined *a priori* but is chosen based on statistical criteria (22). In contrast to previous research using the same dataset (14, 17), which mainly focused on the determinants of EMH and interaction effects, LCA can provide insight into latent subgroups that may be present in the current sample but have not yet been explored. These classes could help researchers and practitioners understand differences and similarities between groups, with implications for future research and the development of tools to foster EMH acceptance.

Therefore, the present study aims to 1) determine how many acceptance prediction classes can be distinguished and 2) describe the classes and differences between classes based on their characteristics (e.g., theoretical orientation, sociodemographic characteristics, perceived advantages and barriers of EMH).

## 2 Methods

### 2.1 Participants and procedures

This analysis is a follow-up to a cross-sectional online study conducted at the University of Zurich during the summer of 2020. Between June and July 2020, participants were recruited using E-mail invitations through well-established educational institutions for psychotherapy in both Germany and the German-speaking region of Switzerland. The survey consisted of 50 questions, and it took participants on average 19.1 minutes to complete (SD = 5.9). In total, outreach efforts were made to 29 institutions in Switzerland and 232 institutions in Germany. However, only a limited number of institutions provided feedback regarding the distribution of the questionnaire, making it impossible to determine the response rate at an institutional level. In total, the survey received 692 visits, out of which 228 participants successfully finished the survey, resulting in a dropout rate of 68.7%. Twelve individuals were omitted from the analysis due to their emergent status as psychotherapist trainees. These participants had solely engaged in the theoretical segment of their training, lacking any clinical experience. Consequently, the final sample size was reduced to 216 participants. The comprehensive outcomes of the original study have been documented separately (14) but can also be found in brief in the introduction of this publication. Ethical safety was provided according to a checklist of the ethics committee of the University of Zurich not requiring any other ethical approval of the ethics committee.

### 2.2 Measures

The survey contained items on sociodemographic characteristics including age, sex, education, country of education (Switzerland or Germany) and theoretical orientation (i.e. cognitive behavioral therapy, depth psychology or psychoanalysis). Acceptance of EMH was operationalized according to the UTAUT (11) and assessed using three items, which were adapted from previous studies (12, 18, 23, 24). A definition of EMH was given to the participants in the beginning of the survey and can be found in the **Supplementary Materials**. UTAUT predictors (Performance Expectancy, Effort Expectancy, Social Influence and Facilitating Conditions) were each assessed with two items. Perceived advantages (time flexibility, simplified information provision, geographic flexibility, and simplified contact maintenance) and barriers (data insecurity, impersonality, irresponsibility, legal concerns, concerns about therapeutic alliance) to EMH were assessed using single items. Three items were adapted from Hennemann et al. (12) and Ebert et al. (25) to assess knowledge about EMH. Experience with EMH was dummy coded into two groups with and without experience. The subjective estimation of evidence on EMH was rated on a visual analogue scale ranging from 1-101. The questionnaire in full can be found in the **Supplementary Materials**.

### 2.3 Statistical analysis

Data was analyzed using IBM SPSS Statistics (Version 27) and R (Version 4.0.0). The LCA computation utilized the poLCA package (26) using the UTAUT predictors as indicators and initially starting with a single-class solution and progressively adding classes. LCA is a popular method for extracting meaningful homogenous subgroups from data (27). Identifying the optimal number of classes is based on indices, such as the Bayesian Information Criterion (BIC), Akaike Information Criterion (AIC), and relative entropy. Notably, the BIC is considered the most robust criterion as it imposes a harsher penalty on the number of parameters than the AIC (22). Smaller AIC and BIC values suggest a more favorable model fit, while greater relative entropy values indicate improved precision concerning the identified classes, with an advisable threshold of 0.8. (22). Upon determining the optimal LCA model based on previously mentioned criteria, individuals were allocated to distinct classes predicated on their posterior class membership probabilities. Differences between the classes were calculated using Chi-Squared tests for count data, Wilcoxon-Test for ordinal variables and t-tests for continuous variables.

## 3 Results

### 3.1 Model selection

**Table 1** shows all tested models and the model-fit criteria. Model 5 showed higher entropy values compared to Model 2, but classes would have been small (around 6% of sample) and the BIC was lowest in Model 2, supporting the two-class solution. In the **Supplementary Material** descriptive statistics of the indicator variables for the two-class solution are also further described and illustrated.

**Table 1:** Evaluating class solutions and model fit criteria.

Model	log-likelihood	resid. df	BIC	aBIC	cAIC	likelihood-ratio	Entropy
Modell 1	-2083.4	184	4338.81	4237.4	4370.81	1906.04	–
**Modell 2**	**-1903.98**	**151**	**4157.35**	**3951.37**	**4222.35**	**1547.2**	**0.808**
Modell 3	-1821.56	118	4169.89	3859.35	4267.89	1382.36	0.82
Modell 4	-1777.28	85	4258.72	3843.6	4389.72	1293.8	0.816
Modell 5	-1736.29	52	4354.13	3834.44	4518.13	1211.83	0.953

Selected model in bold.

### 3.2 Class description

**Figure 1:**
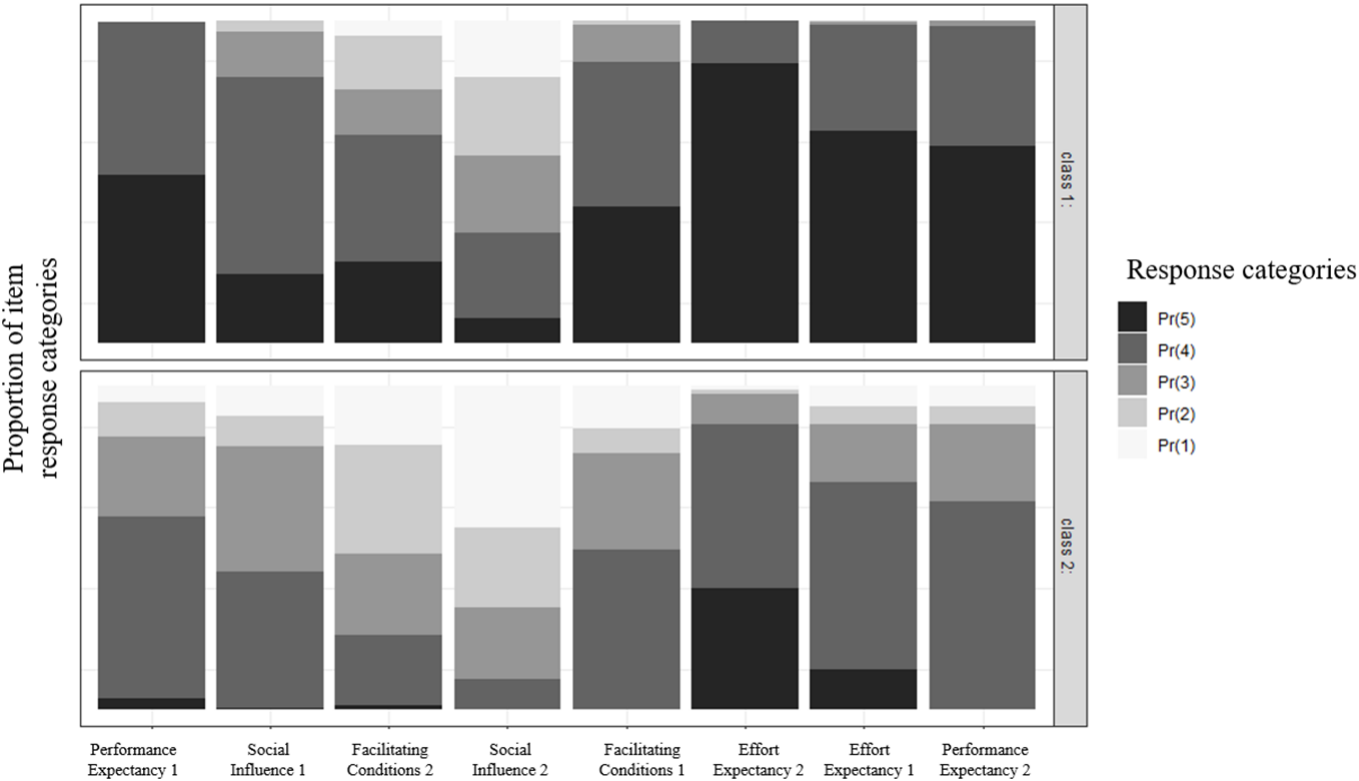
Item ratings across classes.

The first class included the majority of participants (63.4%) and was characterized by very high scores across all UTAUT predictors. The only exception was the second item for Social Influence (“Our patients endorse the use of the following EMH services”), which had the lowest score compared with the other indicator variables. This class was therefore labeled as *highly beneficial factors.* The second class was characterized by moderate expressions across the UTAUT predictors. Next to the second Social Influence item, also one Facilitating Condition item (“The technical equipment of my professional environment is adequate for the implementation of EMH services”) showed lower scores compared to the other predictors. Class 2 was therefore labeled as having *moderately beneficial factors.* Regarding the indicators, the biggest differences between the classes were found in Performance Expectancy and Effort Expectancy (r = 0.67 and r = 0.60). **Figure 1** illustrates, distinctively for class 1 and class 2 the proportion of responses for the eight UTAUT predictors.

While the classes did not differ significantly in terms of age and gender distribution or country of origin, they did differ in therapeutic orientation. Class 1 had significantly (χ2(1) = 5.13, *p* <.05) more participants with a cognitive behavioral orientation compared to Class 2. Descriptive statistics and class comparisons for sociodemographic variables are shown in **Table 2**.

**Table 2:** Sociodemographic variables across classes.

Variable	Total (*n* = 216)	Class 1 (*n* = 137)	Class 2 (*n* = 79)	Statistics
Age, *n* (%)				*χ* ^2^(7) = 9.88, *p* = .20, *V* = 0.21
20-24	5 (2.3)	3 (2.2)	2 (2.5)	
25-29	90 (41.7)	61 (44.5)	29 (36.7)	
30-34	61 (28.2)	42 (30.7)	19 (24.1)	
35-39	28 (13.0)	12 (8.8)	16 (20.3)	
40-44	19 (8.8)	13 (9.5)	6 (7.6)	
45-49	7 (3.2)	3 (2.2)	4 (5.1)	
50-54	1 (0.5)	0 (0.0)	1 (1.3)	
55-59	5 (2.3)	3 (2.2)	2 (2.5)	
Gender, n (%)				*χ* ^2^(1) = 0.02, *p* = .84, *V* = 0.03
Female	191 (88.4)	122 (89.1)	69 (87.3)	
Male	25 (11.6)	15 (10.9)	10 (12.7)	
Country of Training, *n* (%)				*χ* ^2^(1) = 0.02, *p* = .89, *V* = 0.02
Germany	156 (72.2)	98 (71.5)	58 (73.4)	
Switzerland	60 (27.8)	39 (28.5)	21 (26.6)	
Background in, *n* (%)				
Psychology	197 (94.9)	130 (94.9)	67 (84.8)	*χ* ^2^(1) = 5.15, *p* <.05, *V* = 0.17
Medicine	6 (2.2)	3 (2.2)	3 (3.8)	*p*= 0.67 ^a^, *V* = 0.05
Therapeutic Orientation, *n* (%)				
Cognitive/cognitive-behavioural	145 (67.1)	100 (73.0)	45 (57.0)	*χ* ^2^(1) = 5.13, *p* <.05, *V* = 0.16
Psychodynamic/ psychoanalysis	35 (16.2)	18 (13.1)	17 (21.5)	*χ* ^2^(1) = 2.01, *p* = .16, *V* = 0.11
Systemic	27 (6.9)	14(4.4)	13 (11.4)	*χ* ^2^(1) = 1.26, *p* = .26, *V* = 0.09
Humanistic	9 (2.3)	5 (2.2)	4 (2.5)	*p* = 0.73 ^a^, *V* = 0.03
Other	22 (10.2)	13 (9.5)	9 (11.4)	*χ* ^2^(1) = 0.04, *p* = .83, *V* = 0.03

^a^Fischer Exact Test if group size smaller than 5.

Additionally, differences between the classes regarding EMH specific variables were tested. Class 1 scored significantly higher on all perceived advantages and lower on all perceived disadvantages, the only exception being data security where no difference manifested itself. Class 1 also showed significantly (*p* <.01) more experience with and knowledge about EMH. Likewise, the evidence rating and the acceptance of EMH in Class 1 was significantly higher. Descriptive statistics and comparisons across the two classes can be found in **Table 3**.

**Table 3:** EMH variables across classes.

Variable	Total (*n* = 216)	Class 1 (*n* = 137)	Class 2 (*n* = 79)	Statistics
Advantages of EMH, *Mdn* (*SD*)				
Time flexibility	4.00 (1.03)	4.00 (0.97)	3.00 (1.06)	*Z = 3.67*, *p* <.01, *r = 0.25*
Simplified information provision	4.00 (0.90)	4.00 (0.71)	4.00 (1.04)	*Z = 5.01*, *p* <.01, *r = 0.34*
Geographic flexibility	4.00 (0.87)	5.00 (0.69)	4.00 (0.93)	*Z = 6.59*, *p* <.01, *r = 0.44*
Simplified contact maintenance	4.00 (1.14)	4.00 (1.00)	3.00 (1.10)	*Z* = 6.14, *p* <.01, *r = 0.42*
Barriers of EMH, *Mdn* (*SD*)				
Data insecurity	4.00 (1.08)	4.00 (1.11)	4.00 (1.02)	*Z* = 6.14, *p* =.57, *r = 0.04*
Impersonality	3.00 (1.06)	3.00 (1.04)	4.00 (0.90)	*Z* = -5.16, *p* <.01, *r = 0.35*
Irresponsibility	4.00 (1.07)	4.00 (1.10)	4.00 (0.98)	*Z* = -2.46, *p* <.05, *r = 0.17*
Legal concerns	3.00 (1.18)	3.00 (1.22)	4.00 (1.00)	*Z* = -3.76, *p* <.01, *r = 0.26*
Concerns about therapeutic alliance	4.00 (1.12)	4.00 (1.17)	4.00 (0.85)	*Z* = -4.34, *p* <.01, *r = 0.30*

EMH Knowledge, *Mdn* (*SD*)	3.66 (1.12)	4.00 (0.72)	3.33 (0.91)	*Z = 5.65*, *p* <.01, *r = 0.38*
EMH Experience yes, *n* (%)	121 (56.0)	84 (61.3)	42 (46.8)	*χ* ^2^(1) = 3.70, *p* = .05, *V* = 0.14
EMH Evidence rating, *M* (*SD*)	53.56 (24.90)	59.8 (22.55)	42.75 (25.19)	*t* = 4.98, *p* <.01, *D* = 0.73
EMH Acceptance, *Mdn* (*SD*)	3.66 (1.12)	4.00 (1.00)	2.67 (1.03)	*Z = 6.25*, *p* <.01, *r = 0.43*

## 4 Discussion

Our study showed that two classes can be distinguished when using the UTAUT predictors as indicators for the LCA. The first class showed high levels on all UTAUT predictors, the second class revealed moderate levels on the UTAUT predictors, no class showed particularly low scores on the UTAUT predictors, which is in line with previous research (28). The largest differences between classes were found in Performance Expectancy and Effort Expectancy of EMH, which highlights the fact that these aspects would require special attention when developing interventions to foster acceptance. The two classes also revealed differences, regarding the acceptance of EMH, the estimation of evidence, knowledge, and experience. Previous studies have already highlighted the fact that knowledge and experience are positively associated with higher acceptance (10, 12, 25, 29, 30), so it is unsurprising that Class one had higher acceptance scores, more knowledge about and more experience with EMH. This suggests potential directions for future research and underscores key areas for enhancing acceptance facilitating interventions (31).

It is worth mentioning that no class-difference arose among the high scores on data security as a barrier, which emphasizes that this aspect needs to be addressed by either training institutions or the developers of EMH independently of the class membership. Additionally, the fact that Class 2 scored lowest on one of the Facilitating Condition Items (“The technical equipment of my professional environment is adequate for the implementation of EMH services”) highlights the need for workplaces to invest in technology and technical equipment if EMH is planned to be implemented in routine care. Due to the low scores on item 2 of Social Influence (“Our patients endorse the use of EMH services”) it became evident for both classes that the patient’s perspective plays an important role and that the acceptance of PiT needs to be addressed in the clinical context as well.

So far, no other study has tried to build subgroups among PiT focusing on the acceptance of EMH using a LCA. There have been few studies, focusing on patients, that tried to find subgroup-specific differences for established groups (e.g., gender, education) or used median-splits to artificially build subgroups (18, 20). Hennemann et al. (18) showed that acceptance significantly differed between age groups, yielding a significantly higher acceptance score in the youngest quartile. They also found differences in acceptance regarding prior EMH use and higher educational status. Compared with our classes, we did not find any significant difference regarding age, but in line with Hennemann et al. (18) more prior experience was also found in Class 1, which also had a higher acceptance score. Interestingly the theoretical orientation of the PiT was distributed unevenly across the two classes, with significantly more PiT with a cognitive behavioral orientation in Class 1. This suggests that EMH has a distinct role in the training of cognitive behavioral therapists, which most likely can be attributed to the fact that many EMH programs are rooted in cognitive behavioral principles (32). However, due to small numbers of PiT from humanistic or systemic orientations, the previously contrasting results regarding orientation and acceptance were not observable in our sample (33).

LCA was applied in this study to explore whether there were underlying homogenous subgroups among the previously heterogenous sample of PiT. It is surprising that no other study has applied LCA in the field of acceptance of EMH while it has been used to find subgroups for other interventions (34), measurements (35) and particularly often in the field of finding sub-groups in patient populations (36, 37). Thus, we conclude it is a big strength of this study to compute multiple class solutions and not rely on using a median split.

### 4.1 Limitations and future research

We encourage other researchers to validate the classes presented here using larger samples, since we were unable to conduct any validation of the two-class solution as our sample size would have been decreased too much. Thus, it would be worth exploring whether a two-class solution still emerges in larger samples, especially if they are more heterogenous in theoretical orientation of the PiT. In our study the 5-class solution showed the highest entropy. However, due to excessively small class sizes, resulting in a low-subject-to-estimated-parameter ratio that is likely to produce unstable results and since the highest entropy may not necessarily represent the best fitting model but possibly an overfit model, it was not investigated further (38, 39). It is also worth mentioning that even though some statistical differences between the classes could be observed, further research is required to determine to what extend those differences are meaningful for practical implications (e.g., interventions to foster acceptance of EMH). One possibility would be to assess other variables such as EMH literacy or internet usage of PiT to gain a more detailed picture. Another limitation to note is that the presented results are descriptive in nature, precluding causal interpretations. It also needs to be added that the internal consistency for the two-item subscales was not calculated to avoid underestimating of the true reliability (40). However, it is a limitation of this study relying on two-item scales to assess several constructs. Lastly, this has been a secondary analysis with data being collected during the Covid-Pandemic and it remains unclear how much practice and acceptance has changed in the meantime.

## 5 Conclusion

The value of this publication lies in the successful identification of two classes of PiT using Latent Class Analysis, based on the UTAUT predictors. Classes showed some distinct features in respect to the indicator variables, especially regarding Performance and Effort Expectancy. Our study revealed that while sociodemographic characteristics did not differ between the classes, knowledge, estimation of evidence, experience and acceptance did. Also, we found that most perceived barriers were rated higher and all advantages rated lower in the *moderately beneficial factors* class. In the future, latent class analysis could help to identify subgroups and highlight possible starting points to foster acceptance of EMH.

## Data availability statement

‘The original contributions presented in the study are included in the article/**Supplementary Material**. Further inquiries can be directed to the corresponding author.

## Ethics statement

The studies involving humans were approved by the ethics committee of the University of Zurich via a checklist indicating that the ethical safety of the study was guaranteed, no further approval of the ethics committee was necessary. The studies were conducted in accordance with the local legislation and institutional requirements. The participants provided their written informed consent to participate in this study.

## Author contributions

RS: Conceptualization, Data curation, Formal analysis, Methodology, Visualization, Writing – original draft. MS: Formal analysis, Supervision, Visualization, Writing – review & editing. MD: Conceptualization, Methodology, Supervision, Writing – review & editing.
