# Supplementary material for: Exploring subgroups of acceptance prediction for e-mental health among psychotherapists-in-training: a latent class analysis
Source: Front Psychiatry. 2024 Mar 14;15:1296449. doi: 10.3389/fpsyt.2024.1296449 (PMC10973105; doi:10.3389/fpsyt.2024.1296449)
Supplement: Supplementary file 1 [file DataSheet_1.docx]

Supplementary Material

What characterizes Psychotherapists-in-Training who strongly accept E-Mental Health from those who do not? A Latent Class Analysis

Robert Staeck, Miriam Stüble, Marie Drüge^*^

*** Correspondence:** Dr. Marie Drüge: [Marie.Druege@psychologie.uzh.ch](mailto:Marie.Druege@psychologie.uzh.ch)

# Supplementary Figures and Tables

## Supplementary Figures

**Supplementary Figure 1.** **Indicator variables across classes**


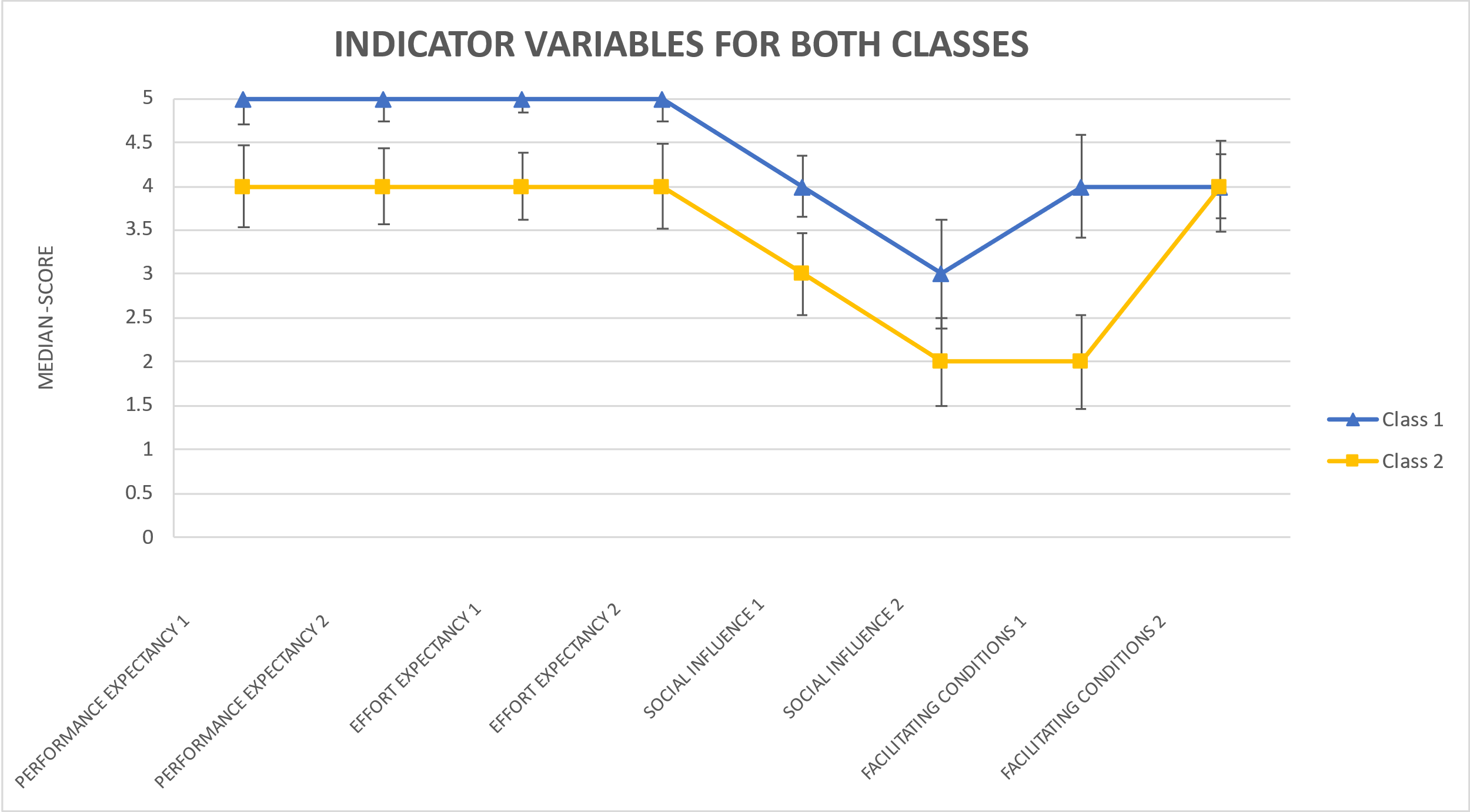


## Supplementary Tables

**Supplementary Table 1. Indicators across classes**

| Variable, Mdn (SD) | Total  (*n* = 216) | Class 1  (*n* = 137) | Class 2  (*n* = 79) | Statistics |
| --- | --- | --- | --- | --- |
|  |  |  |  |  |
| Performance Expectancy 1 | 4.00 (0.88) | 5.00 (0.58) | 4.00 (0.92) | *Z* = 8.92, *p* < .01,  *r* = 0.61 |
| Performance Expectancy 2 | 4.00 (0.86) | 5.00 (0.53) | 4.00 (0.86) | *Z* = 9.78, *p* < .01,  *r* = 0.67 |
| Effort Expectancy 1 | 5.00 (0.62) | 5.00 (0.32) | 4.00 (0.76) | *Z* = 9.78, *p* < .01,  *r* = 0.67 |
| Effort Expectancy 2 | 5.00 (0.87) | 5.00 (0.53) | 4.00 (0.96) | *Z* = 8.86, *p* < .01,  *r* = 0.60 |
| Social Influence 1 | 4.00 (0.89) | 4.00 (0.70) | 3.00 (0.93) | *Z* = 6.84, *p* < .01,  *r* = 0. 47 |
| Social Influence 2 | 2.00 (1.23) | 3.00 (1.24) | 2.00 (1.01) | *Z* = 4.70, *p* < .01,  *r* = 0.32 |
| Facilitating Conditions 1 | 3.00 (1.25) | 4.00 (1.16) | 2.00 (1.07) | *Z* = 6.40, *p* < .01,  *r* = 0.46 |
| Facilitating Conditions 2 | 4.00 (1.00) | 4.00 (0.73) | 4.00 (1.03) | *Z* = 7.87, *p* < .01,  *r* = 0.54 |

**Supplementary Table 2. Translation and Source of Items**

| **Construct** | **Source** | **Items (rating scale)** |
| --- | --- | --- |
| Acceptance | Adapted from Hennemann et al. 2017, Venkatesh et al. 2003; Elfeddali et al. 2013 | I could imagine including the following EMH services in my work. (1-5)*  I intend to try out the following EMH service in my work within the next year. (1-5)*  How high is your intention to use the following  EMH service in your work ever? (0-100)** |
| UTAUT predictors | Adapted from Hennemann et al. 2017; Venkatesh et al. 2003 | *Performance expectancy (1-5)*:*  The following EMH service would be a useful extension to existing treatment measures.  The following EMH services could improve  patient’s health status.  *Effort expectancy (1-5)*:*  I would find the following EMH service easy to use and to understand.  I would easily learn to operate the following EMH service.  *Social influence (1-5)*:*  My colleagues would approve the use of the following EMH service.  Our patients endorse the use of the following EMH services.  *Facilitating conditions (1-5)*:*  I have the necessary technical preconditions for using the following EMH services.  The technical equipment of my professional environment is adequate for the  implementation of EMH services. |
| Application purposes | Self-constructed | Which of the following EMH services would you use for prevention? (1-5)*  Which of the following EMH services would you use for aftercare? (1-5)*  Which of the following EMH services would you use as an addition to therapy? (1-5)* Which of the following EMH services would  you use as a substitute for therapy? (1-5)* |
| Advantages | Adapted from Hennemann et al. 2016; Bendelin et al. 2011; Berger 2015; Wells et al. 2007 | I believe the following EMH services would simplify the provision of information and exercises. (1-5)*  Regarding the following EMH services, I think it is beneficial that they are flexibly accessible. (1-5)*  The following EMH services are especially helpful for patients living in rural areas as  they facilitate the access. (1-5)*  The following EMH services allow me to maintain contact with my patients. (1-5)* |
| Barriers | Adapted from Hennemann et al. 2016; Bendelin et al. 2011; Berger 2015; Wells et al. 2007 | Regarding the following EMH services, I have concerns about data security. (1-5)*  The following EMH services are too impersonal and not interactive enough. (1- 5)*  Regarding the following EMH services, I have concerns about my responsibility as a psychotherapist towards my patients (e.g., in emergency situations). (1-5)*  Regarding the following EMH services, I have legal concerns (e.g., because I am not sufficiently informed about the current legal situation). (1-5)*  Regarding the following EMH services, I have concerns about not being able to build a viable therapeutic relationship. (1-5)* |
| Knowledge about  EMH | Adapted from Hennemann et al. 2017; Ebert et al., 2015 | I have an idea of what the following EMH services are. (1-5)*  I know what I can expect when using the following EMH services as a therapeutic tool. (1-5)*  I have some knowledge on the following EMH services. (1-5)* |
| EMH experience | Adapted from Hennemann et al. 2017; Eichenberg et al. 2013 | In percentage, how much do you already use the following EMH services in your therapeutic work? (0-100)***   - for psychotherapy: how much percentage of your psychotherapy sessions did you execute by phone or videoconference, respectively? - for VR: How many patients do you serve with VR? - for (un-)guided programs: how much percentage of your patients do you serve   with e-health programs? (or did you recommend such a program?) |
| Evidence assessment | Self-constructed | How strong would you rate the scientific evidence base of the following EMH services (0-100)**** |

*Note.* Original Descriptions were in German, adapted from Braun et al., (2022)

*rating scale: (1) *totally disagree* to (5) *totally agree*

**rating scale: (1) *no intention* to (100) *very strong intention*

***rating scale: (1) *never* to (100) *always*

****rating scale: (1) *very low* to (100) *very high*

**Supplementary Table 3. Introduction and Information to EMH**

| EMH | E-Mental Health is a general term for information and communication technologies (both devices such as mobile phones, computers and programs such as apps). These can be supportive and help to improve a person's mental health. There is a range of very different E-mental health technologies. |
| --- | --- |

*Note.* Original Descriptions were in German
